# Clinical Benefits of Conversion Surgery for Unresectable Pancreatic Ductal Adenocarcinoma: A Single-Institution, Retrospective Analysis

**DOI:** 10.3390/cancers13051057

**Published:** 2021-03-02

**Authors:** Yuko Mataki, Hiroshi Kurahara, Tetsuya Idichi, Kiyonori Tanoue, Yuto Hozaka, Yota Kawasaki, Satoshi Iino, Kosei Maemura, Hiroyuki Shinchi, Takao Ohtsuka

**Affiliations:** 1 Department of Digestive Surgery, Breast and Thyroid Surgery, Graduate School of Medical and Dental Sciences, Kagoshima University, 8-35-1, Kagoshima 890-8520, Japan; h-krhr@m3.kufm.kagoshima-u.ac.jp (H.K.); k3352693@kadai.jp (T.I.); wilson@m.kufm.kagoshima-u.ac.jp (K.T.); yhozaka@kufm.kagoshima-u.ac.jp (Y.H.); k5968102@kadai.jp (Y.K.); iino@m2.kufm.kagoshima-u.ac.jp (S.I.); takao-o@kufm.kagoshima-u.ac.jp (T.O.); 2 Department of Digestive Surgery, Kagoshima Principal Hospital, Kagoshima 890-0055, Japan; maemura-k01@kch.kagoshima.jp; 3 Department of health sciences, School of Medicine, Kagoshima University, Kagoshima 890-8520, Japan; shinchi@m.kufm.kagoshima-u.ac.jp

**Keywords:** unresectable pancreatic ductal adenocarcinoma, conversion surgery, chemoradiotherapy

## Abstract

**Simple Summary:**

Pancreatic ductal adenocarcinoma (PDAC) is a dismal disorder, but conversion surgery (CS) has provided possibilities of better prognosis for unresectable (UR-)PDAC. We retrospectively investigated the clinical benefits of CS in patients with UR-PDAC. We performed CS in 20 of the 398 UR-PDAC cases between 2006 and 2019(5.1%). Compared the overall survival (OS) period among patients undergoing CS, resectable (R), borderline resectable (BR), unresectable locally advanced cancer (UR-LA), and unresectable cancer with distant metastasis (UR-M) groups, the CS group had significantly better survival than R, BR, UR-LA, and UR-M groups (73.7, 32.7, 22.7, 15.7, and 8.8 months, respectively). Moreover, multivariate analysis revealed the presence of chemoraiotherapy and partial response/complete response in the Response Evaluation Criteria in Solid Tumors (RECIST) were statistically significant prognostic factors for OS among patients undergoing CS (*p* = 0.004 and 0.03, respectively). This study highlights importance of multidisciplinary treatment including CS for patients with UR-PDAC.

**Abstract:**

Background: Unresectable pancreatic ductal adenocarcinoma (UR-PDAC) has a poor prognosis. Conversion surgery is considered a promising strategy for improving the prognosis of UR-PDAC. This study aimed to investigate the clinical benefits of conversion surgery in patients with UR-PDAC. Methods: We retrospectively evaluated patients with PDAC who were referred to our department for possible surgical resection between January 2006 and December 2019. Conversion surgery was performed only in patients with UR-PDAC who could expect R0 resection. We analyzed the prognostic factors for overall survival among patients who underwent conversion surgery. Results: Overall, 638 patients with advanced pancreatic cancer were enrolled in this study. According to resectability, resectable cancer (R) was present in 180 patients, borderline resectable cancer (BR) was present in 60 patients, unresectable locally advanced cancer (UR-LA) was present in 252 patients, and unresectable cancer with distant metastasis (UR-M) was present in 146 patients. Conversion surgery was performed in 20 of the 398 UR cases (5.1%). The median period between the initial therapy and conversion surgery was 15.5 months. According to the Response Evaluation Criteria in Solid Tumors (RECIST) evaluation, the treatment response was CR in one patient, PR in 13, SD in five, and PD in one. Downstaging was pathologically determined in all cases. According to the Evans grading system, grade I was observed in four patients (20%), grade IIb was observed in seven (35%), III was observed in seven (35%), and IV was observed in two (10%). We compared the overall survival period from initial treatment among patients undergoing conversion surgery; the median overall survival durations in the conversion surgery, R, BR, UR-LA, and UR-M groups were 73.7, 32.7, 22.7, 15.7, and 8.8 months, respectively. Multivariate analysis revealed that the presence or absence of chemoradiotherapy (CRT) and the RECIST partial response (PR)/complete response (CR) for the main tumor were statistically significant prognostic factors for overall survival among patients undergoing conversion surgery (*p* = 0.004 and 0.03, respectively). Conclusion: In UR-PDAC, it is important to perform multidisciplinary treatment, including CRT with conversion surgery.

## 1. Introduction

Pancreatic ductal adenocarcinoma (PDAC) is a very dismal disease with a poor prognosis among malignant tumors in Japan [1]. Even in the United States, PDAC is the fourth leading cause of cancer-related deaths [2]. This disease has a 5-year survival rate of approximately 10%. Surgical resection is the only potentially curative therapy, but only 10–20% of patients with resectable PDAC are classified at the time of the initial diagnosis [3]. When we select the methodology of treatment for a patient with PDAC, the appropriate treatment is likely to decided based on the resectable classification rather than the stage classification. The National Comprehensive Cancer Network (NCCN) has proposed a resectable classification for PDAC and recommended optimal therapy based on each resectability [4]. Among them, pancreatic cancer patients with unresectable locally advanced cancer (UR-LA) and unresectable cancer with metastasis (UR-M) are recommended to receive chemotherapy if their performance status is good. Combination chemotherapy with fluorouracil, leucovorin, irinotecan, and oxaliplatin (FOLFIRINOX) or modified (mFOLFIRINOX), chemoradiation, gemcitabine plus nab-paclitaxel (PTX), and/or chemoradiation are listed as candidate regimens for chemotherapy. The Japanese Pancreas Society designed new and definite resectability criteria in 2019 based on the NCCN guideline [5]. Regarding resectable advanced pancreatic cancer, a prospective randomized trial in Japan compared surgical resection and chemoradiotherapy (CRT) after laparotomy was used to identify the localized pancreatic cancer invading the surrounding vasculature; this study showed that patients who underwent surgical resection had significantly longer survival than those treated with CRT alone [6].

In recent reports, multidisciplinary treatments, including surgical resection and chemotherapy or chemoradiation therapy, have improved the survival of patients with PDAC [7,8,9]. As for patients with resectable (R) PDAC, adjuvant chemotherapy has become standard even after curative resection [10,11]. Moreover, many studies have reported the effectiveness of neoadjuvant chemotherapy. In recent years, patients with UR-PDAC have been able to achieve treatment effects to be converted to surgical resection. This surgical strategy is called “conversion surgery”. Therefore, several reports about conversion surgery have been published in selected patients with initially UR-PDAC, and the prognostic effect of conversion surgery has been reported [12,13,14,15,16,17,18,19,20,21,22]. However, it is unclear whether conversion surgery with a high treatment effect is truly advantageous in patients with UR-PDAC.

In this study, we aimed to clarify the clinical benefits and important factors of conversion surgery for patients with UR-PDAC who underwent CRT.

## 2. Methods

### 2.1. Ethics Statements

All research performed in studies involving human participants were according to discipline of the institutional research committee and with the 1964 Declaration of Helsinki. Written informed consent was obtained from all study participants.

### 2.2. Study Design and Patient Population

This retrospective study was performed using data from a prospective database. All patients with PDAC treated at the Department of Digestive Surgery, Breast and Thyroid Surgery, Kagoshima University between January 2006 and December 2019 were enrolled in our study. PDAC was diagnosed by cytology or pathology through endoscopic retrograde cholangiopancreatography or endoscopic ultrasound-guided fine-needle aspiration. PDAC progression was diagnosed using multidetector-row computed tomography, ethoxybenzyl-magnetic resonance imaging, and fluorine-18-2-deoxy-D-glucose positron emission tomography. All patients were divided into resectable cancer (R), borderline resectable cancer (BR), UR-LA, and UR-M, which was defined according to the NCCN Clinical Practice Guidelines version 2 from 2020 [4].

### 2.3. Therapy Method

The gemcitabine and S-1 (GS) regimen is as follows, that the daily dosage of S-1 chemotherapy was 60 mg/m^2^ combined with 1000 mg/m^2^ gemcitabine. Patients received gemcitabine intravenously on days 8 and 15 of a 21-day cycle and oral S-1, twice daily on days 1–14. The GEM plus nab-PTX regimen consisted of gemcitabine 1000 mg/m^2^ and nab-PTX 125 mg/m^2^ administered on days 1, 8, and 15, every 28 days. The modified FOLFIRINOX (m-FOLFIRINOX) regimen used in our study was the same as that of a Japanese phase II study and consisted of oxaliplatin at a dose of 85 mg/m^2^, administered as a 2-h intravenous infusion, immediately followed by leucovorin at a dose of 200 mg/m^2^, administered as a 2-h intravenous infusion, with the addition, after 30 min of irinotecan at a dose of 180 mg/m^2^, administered as a 90-min intravenous infusion through a bypass line. This treatment was immediately followed by fluorouracil at a dose of 400 mg/m^2^ administered via intravenous bolus, followed by a continuous intravenous infusion of 2400 mg/m^2^ over a 46-h period, every 2 weeks. CRT regimens included hyper-fractionated accelerated radiotherapy administered with S-1 at 80 mg/m^2^ for the first 21 days. A total of 50–58 Gy was administered in 40 fractions over 4 weeks. At 1 month after CRT completion, S-1 was administered for 2 weeks, followed by a 2 weeks period.

The GEM plus nab-PTX and FOLFIRINOX regimens were approved in Japan for the treatment of UR-PC in December 2014, and these regimens have been utilized since then.

### 2.4. Conversion Surgery

We discussed and decided the indication for conversion surgery for individual UR-PDAC patients at a multidisciplinary conference of pancreatic surgeons, medical oncologists, radiologists, and clinical pathologists. Among UR-PDAC patients who responded to various therapies, conversion surgery was permitted for only those who met the following conditions: patients showing adequate reduction of the main tumor, enabling complete removal inclusive of the major vessels and metastatic site, those with at least several months of local control, those with no metastasis, or those with controllable metastasis by surgical resection.

### 2.5. Adjuvant Chemotherapy

We basically consider adjuvant therapy after conversion surgery if the patient is in good condition. Furthermore, if new lesions are operable after conversion surgery, we consider additional resection for the lesions.

### 2.6. Assessment

The clinical treatment effect was assessed using RECIST version 1.1 [23], and the histologic assessment of the extent of response was evaluated using the Evans grading system [24]. R0 was defined as pathologically margin free in the resected specimen.

The Clavien–Dindo classification was used to evaluate postoperative complications [25]. Mortality was defined as death within 90 days after surgery.

### 2.7. Statistical Analysis

Associations between different categorical variables were properly assessed using the chi-square or Fisher exact test. Survival curves were estimated using the Kaplan–Meier method and analyzed using the log-rank test. Overall survival was calculated as the interval between initial treatment and death due to any cause (including death from other diseases), whereas progression-free survival was calculated as the interval between initial treatment and disease progression. The univariate Cox proportional hazard models were used to estimate the independent significant factors for the overall survival of patients with UR-PDAC. Relative risks were expressed as adjusted hazard ratios and corresponding 95% confidence intervals. Valuables with a *p*-value of < 0.05 on univariate analysis were calculated into the multivariate model. A *p*-value of < 0.05 was considered to be statistically significant. All statistical analyses were performed using SigmaPlot, version 12.5 for Windows (HULINKS, Inc., Tokyo, Japan).

## 3. Results

### 3.1. Patient Characteristics

During the above observation period, 638 patients were included. R was present in 180 patients, BR was present in 60, UR-LA was present in 252, and UR-M was present in 146. Overall, 398 consecutive patients with unresected pancreatic cancer were studied in terms of their unresectable status. Conversion surgery was performed in 20 patients (20/398, 5.0%; UR-LA, *n* = 9; UR-M, *n* = 11). The clinical characteristics of patients with and without conversion surgery are compared in Table 1.

There were no statistically significant differences in age, sex, the tumor location (pancreatic head versus (vs.) pancreatic body and tail), unresectability status (UR-LA vs. UR-M), serum carcinoembryonic antigen (CEA) level, cancer antigen 19-9 (CA19-9) level, tumor size, and T-, N-, and M-categories according to the Tumor-Node-Metastasis (TNM) classification before treatment between patients with and without conversion surgery.

**Figure 1:**
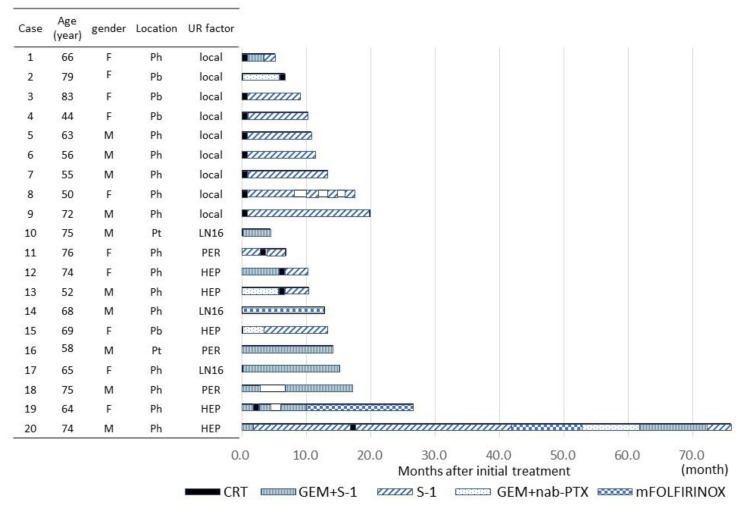
Clinical characteristics of the 20 patients who underwent conversion surgery including therapy before operation. Abbreviations: UR-LA, unresectable locally advanced cancer; UR-M, unresectable cancer with metastasis; M, male; F, female; Ph, pancreatic head; Pb, pancreatic body; Pt, pancreatic tail; LN16, para-aortic lymph node metastasis; PER, peritoneal disseminations; HEP, hepatic metastases; CRT, chemoradiotherapy; GEM, gemcitabine; S-1, oral administration of S-1; nabPXL, nab-paclitaxel; mFOLFIRINOX, modified FOLFIRINOX (combination chemotherapy with fluorouracil, leucovorin, irinotecan, and oxaliplatin).

**Figure 2:**
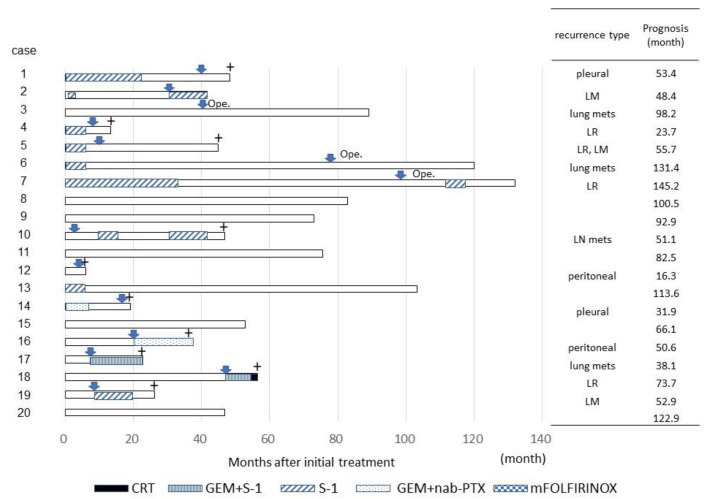
Clinical characteristics of the 20 patients who underwent conversion surgery including therapy after operation. Abbreviations: pleural, pleural disseminations; LM, liver metastasis; lung mets, lung metastasis; LR, local recurrence; peritoneal, peritoneal disseminations; directing arrow, recurrent or metastatic period; Ope, additional operation; +, period of death.

The clinical characteristics of the 20 patients who underwent conversion surgery are shown in Figure 1 and Figure 2 and Table 2 and Table 3.

Among the 11 patients with UR-M, the type of metastasis at the time of initial diagnosis was hepatic metastasis in five patients, peritoneal dissemination in three, and para-aortic lymph node metastasis in three. Histological confirmation of the metastatic lesions was made in all patients with peritoneal disseminations and para-aortic lymph node metastasis, but this was not the case in three of five patients with liver metastasis.

The periods between initial treatment and conversion surgery were a median of 10.8 (range, 5.1–19.9) months in patients with UR-LA and median of 13.3 (range, 4.4–75.9) months in those with UR-M. There were no significant differences in the period between the groups.

The methods of therapy until conversion surgery varied, but CRT was performed in all patients with UR-LA. Among them, CRT was selected as the initial therapy in all patients except one (case 2, Figure 1), and CRT was performed after gemcitabine plus nab-PTX therapy as induction chemotherapy in the remaining case (case 2, Figure 1).

Chemotherapy of various types was performed in patients with UR-M, and additional CRT was carried out to perform local control in five of these cases. According to the RECIST evaluation, the treatment response after various therapies was complete response (CR) in one patient, partial response (PR) in 13, stable disease (SD) in five, and progressive disease (PD) in one.

The CA19-9 level at the time of conversion surgery was significantly lower than that at the time of initial diagnosis (median, 16.4 U/mL (range, 0.6–99) vs. median, 156 U/mL (range 0.6–1985), *p =* 0.0065), but the CEA level was not significantly different at the time between the initial diagnosis and conversion surgery (*p =* 0.418). The size of the main tumor at the time of conversion surgery was significantly smaller than that at the time of diagnosis (median, 13 mm (range, 0–50) vs. 30 mm (range, 18–50), *p <* 0.001). In cases 6, 17, and 19, the CEA level, CA19-9 level, and size of the main tumor after each therapy were reduced compared with those in the period before initial therapy, respectively (Table 2).

As for the operative method, substomach-preserving pancreaticoduodenectomy was performed in 13 patients, including concomitant portal vein resection (PVR) in six patients and hepatic resection (HPR) in one patient. Distal pancreatectomy and total pancreatectomy with PVR and HPR were performed in six patients and one patient, respectively (Table 2). The median operative time and volume of blood loss were 575 (range, 352–919) min and 1150 (range, 90–3600) mL, respectively. The median postoperative stay was 13 (range, 8–50) days. Major complications in the postoperative period (Clavien–Dindo classification class IIIa) occurred in three patients with chylous ascites, interstitial pneumonia, and intraabdominal abscess (cases 1, 3, and 14, respectively; Table 2); those complications caused patients’ postoperative hospital stay to exceed 1 month. No perioperative mortality was observed. R0 resection of the main tumor was performed in all patients. Compared with before and after various therapies, clinical stage was downstaged in 13 cases, and the pathological stage was downstaged in 19 cases. Pathologically, tubular adenocarcinoma with high differentiation and tubular adenocarcinoma with moderate differentiation were the pathological forms of the residual tumor in six and eight cases, respectively. According to the Evans grading system, grade I was observed in four patients (20%), grade IIb in seven (35%), III in seven (35%), and IV in two (10%). A pathological CR was observed in two patients (cases 10 and 16, Table 2). The pathological assessment was difficult to perform in one case because of a few residual cancer cells (case 20, Table 2). In two patients who underwent hepatic resection combined with pancreatectomy, one patient had residual cancer cells in the resected liver.

### 3.2. Adjuvant Chemotherapy

Only eight patients (40%) received postoperative adjuvant chemotherapy, and S-1 was administered to all those patients. The reasons why 12 patients did not receive adjuvant chemotherapy were poorer performance status, severe perioperative complications, elderly age, and patient unwillingness. Recurrence occurred in 14 patients (70%) who underwent conversion surgery and received various therapies. The recurrence type varied: dissemination occurred in four patients, local recurrence, including metachronous double cancer, in three, liver metastasis in three, lung metastasis in three, and lymph node metastasis in one. Two patients with lung metastasis and one with metachronous double cancer of the remnant pancreas underwent additional resection of the metastatic lesion at 41, 78, and 97 months after conversion surgery, respectively (cases 3, 6 and 7; Figure 2).

**Figure 3:**
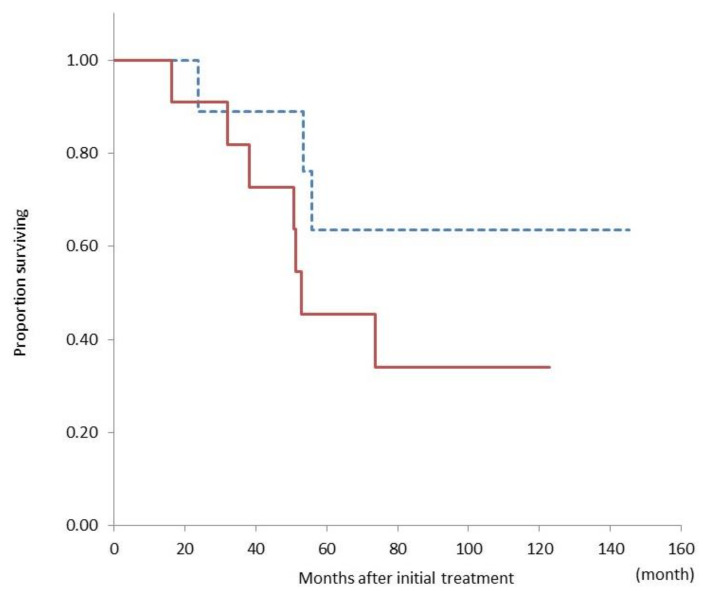
Comparison of the overall survival curves between patients with unresectable locally advanced cancer (dashed line, *n* = 9) and unresectable cancer with metastasis (solid line, *n* = 11) who underwent conversion surgery. There is no significant difference in overall survival between the groups (*p =* 0.20).

We compared overall survival from the period of initial treatment between patients who underwent conversion surgery with UR-LA and those with UR-M and found no significant difference between these patient groups (median not reached vs. 52 months, *p =* 0.20) (Figure 3).

**Figure 4:**
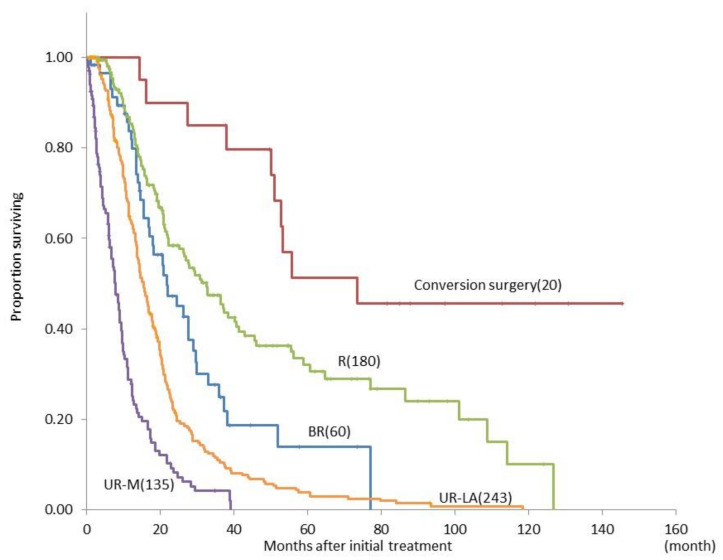
Comparison of the overall survival curves between patients with pancreatic ductal adenocarcinoma judged as R (*n* = 180), BR (*n* = 60), UR-LA (*n* = 243), or UR-M, (*n* = 135) according to the NCCN guideline, who underwent conversion surgery (*n* = 20). Abbreviations: R, resectable cancer; BR, borderline resectable cancer; UR-LA, unresectable locally advanced cancer; UR-M, unresectable cancer with metastasis; NCCN, National Comprehensive Cancer Network.

Moreover, we compared overall survival from the period from initial treatment between the conversion surgery, R, BR, UR-LA, and UR-M groups according to the NCCN guidelines. The median overall survival durations in the conversion surgery, R, BR, UR-LA, and UR-M groups were 73.7, 32.7, 22.7, 15.7, and 8.8 months, respectively (Figure 4). There were significant differences in the survival period between each group (*p*-value not shown).

By the univariate and multivariate logistic regression analyses, we researched the prognostic factors for overall survival among patients who underwent conversion surgery. Table 4 shows that the presence of CRT and RECIST PR/CR for the main tumor decreased the risk of death relative to the absence of CRT and RECIST SD/PD for the main tumor (*p =* 0.002 and 0.015, respectively). Multivariate analysis revealed that the presence of CRT and RECIST PR/CR for the main tumor was a statistically significant prognostic factor for overall survival among patients undergoing conversion surgery (*p =* 0.004 and 0.03, respectively).

## 4. Discussion

The reports on conversion surgery for locally advanced pancreatic cancer have been increasing since the 2010s [12,13,14,15,16,17,18,19,20,21,22]. According to the recent NCCN guidelines (version 1.2021) [4], surgical resection of locally advanced cancer is a subsequent therapy option for patients with good performance status and no disease progression after first-line therapy. The present retrospective study found that conversion surgery for UR-PDAC after various therapy is relatively safe and feasible, because of no mortality and lower severe morbidity (C-D classification ≥ IIIa was 15% rate), and it may help improve long-term prognosis.

In the current study, the resection rate was only 5.0% (UR-LA, 3.6%; UR-M, 7.5%). However, this value was very low compared with that in previous reports; the reason for the resection rate might depend on policy whether we actively accepted conversion surgery when multidisciplinary treatment was performed for patients with UR-PDAC [16]. Many studies have focused on the effectiveness of conversion surgery, and although patients with UR-PDAC were not candidates for surgery, conversion surgery was performed for only an extremely limited number of patients who were super-responders to palliative therapy. Hackert et al. reported that among 575 patients with locally advanced PDAC receiving neoadjuvant therapy, 292 (50.8%) underwent pancreatic resection, which was a higher resection rate than that in our study [7]. However, the median survival time (MST) after adjuvant surgery was only 15.3 months. In the current study, the median overall survival was 73.7 months, which is better than that in previous reports [13,16,18]. The MST of patients with UR-LA treated with no resection was reported to be 15–17 months, which was similar to that in the present study. The median overall survival durations in PDAC patients with UR-LA and UR-M were 15.7 and 8.8 months, respectively. On the other hand, Lee et al. reported an excellent outcome, showing that 15 of 64 (23.4%) PDAC patients with UR-LA who underwent conversion surgery had an overall survival exceeding 40 months, although they did not clearly describe the indications for surgery [26]. Strict criteria similar to those in our study may lead to lower resectability but longer overall survival as a result of patient selection, so we may need to expand our surgical indication.

Conversion surgery has provided a surprising outcome for patients with UR-PDAC. In particular, the prognosis of patients with UR-PDAC who underwent conversion surgery was significantly better than that of patients with resectable PDAC. Several authors have previously reported the usefulness of conversion surgery in such patients, as well as favorable results on prognosis [19,27,28,29,30]. In the present study, the prognosis of PDAC patients with UR-LA and UR-M who underwent conversion surgery was not significantly different. Among the patients with UR-PDAC who underwent conversion surgery, we identified that RECIST CR/PR after the various treatments was an important prognostic factor by multivariate analysis. It may be important to perform multidisciplinary treatment aimed at tumor shrinkage and possible conversion surgery in order to obtain a better prognosis. However, recurrence and metastasis after conversion surgery were observed in 70% of patients, especially early recurrence within one year after surgery in 30% of patients. Uesaka et al. demonstrated the non-inferiority of S-1 to gemcitabine as adjuvant chemotherapy for pancreatic cancer in terms of overall survival in a randomized trial [10]. Chad et al. reported that adjuvant therapy was not required for all patients with localized pancreatic cancer who had received neoadjuvant therapy; the benefit of adjuvant therapy was limited to those with node-positive disease [31]. In the current study, adjuvant chemotherapy after conversion surgery was performed in only eight (40%) of 20 patients. In other reports, early recurrence after conversion surgery occurred in approximately 30% of cases [32,33]. The conversion surgery tended to have a long operative time and high bleeding volume in the present study, so it was considered a major invasive surgery. Therefore, it may be important to reduce morbidity in surgery as much as possible and to create an environment in which adjuvant chemotherapy can be easily performed. The early recurrence rate should be decreased as much as possible in patients undergoing conversion surgery [34]. Moreover, in the present study, during the follow-up after conversion surgery, additional surgical resection was performed in one patient with double cancer in the remnant pancreas and two patients with single lung metastasis. The surgical resection for multicentric cancer in the remnant pancreas and solitary lung metastasis may have possibilities to provide long-term survival. [35,36].

Satoi et al. previously performed multicenter joint research that focused on conversion surgery of initially unresectable PDAC [13]. This study included 58 PDAC patients (41 with UR-LA and 17 with UR-M). The MST in the conversion surgery group was significantly better than that in the control group (39.7 months vs. 20.8 months, *p* < 0.0001). The optimal timing of adjuvant surgery was 240 days after the initial treatment. Moreover, a multivariate analysis showed that for the adjuvant surgery group, significant favorable factors for overall survival included the dosage above a certain level of gemcitabine, a decrease in tumor markers until conversion surgery, and PR/CR evaluation by RECIST. Michelakos et al. reported that in resected patients with BR/locally advanced PDAC treated with FOLFIRINOX, a preoperative CA19-9 level >100 U/mL and >8 months between diagnosis and surgery predicted a shorter postoperative DFS [33]. Therefore, the optimal period between the initial diagnosis and conversion surgery is controversial.

As for the pathological examination, the Evans grade, which reflects the extent of tumor degeneration or necrosis after chemo(radio)therapy, has been extensively identified as a prognostic factor [19,37,38,39]. Chatterjee et al. reported that among the 223 patients with resectable PDAC who received neoadjuvant chemoradiation and had pancreaticoduodenectomy, pCR (grade IV, 2.7%) or minimal residual tumor (grade III, 16.1%) in posttreatment specimens of pancreaticoduodenectomy correlated with better survival [40]. Histologically, grades IIb, III, and IV, i.e., destroyed areas over 50%, were observed in the main tumor in 16 of the 20 patients who underwent conversion surgery in the present study. Moreover, the residual tumor tended to contain a wide range of highly differentiated cells, so this result suggests that cells with poorly differentiation may be highly effective for chemo(radio-)therapy.

In the present study of patients with UR-PDAC who underwent conversion surgery, we identified important prognostic factors from initial treatment in multivariate analysis: the use of chemoradiotherapy and RECIST CR/PR after various treatments. Of the treatment for UR-PDAC, radiation therapy combined with systemic chemotherapy is one of the recommended therapies in the NCCN [4] and Japanese guidelines [5]. In our institution, we reported the usefulness of the combination therapy with S-1 and radiation in patients with UR-PDAC; it is a well-tolerated regimen that can be recommended as an effective treatment in prospective phase II trials, and it showed favorable survival with a median survival time of 14.3 months [41]. Jang et al. reported on the benefit of neoadjuvant treatment in patients with BR-PDAC, and gemcitabine-based neoadjuvant chemoradiation provided oncological benefits compared to upfront surgery by the prospective randomized controlled trial [42]. A recent LAP-07 trial comparing chemotherapy and CRT for locally advanced PDAC failed to show any survival benefit of CRT [43]. However, CRT was associated with a decreased local progression rate and no increase in grade 3 or 4 toxicities. This result may indicate that CRT has potential as a more useful method in conversion surgery.

In the present study, we identified no significant difference in overall survival between patients who underwent conversion surgery with UR-LA and UR-M. Yanagimoto et al. reported no significant differences between the two groups, which is similar to our study finding [18]. There are few reports of surgical resection of pancreatic resection with synchronous metastases. Wright et al. reported 23 cases of surgical resection of stage IV pancreatic cancer after a favorable response to systematic chemotherapy [27]. The sites of metastasis included the liver (*n* = 16), lung (*n* = 6), and peritoneum (*n* = 2). The treated patients with stage IV disease were 1147 cases in all, so the resection rate was only 2.0%. The MST from the initial diagnosis was 34.1 months. Frigerio et al. reported that 24 (4.5%) of 535 patients diagnosed as pancreatic cancer with liver metastasis underwent surgical resection of the primary site and hepatic resection [44]. The MST after diagnosis in the study was 56 months. The limited number of patients with distant metastasis (super-responders) accounted for less than 5% of PDAC patients with UR-M. The effectiveness of conversion surgery for metastatic PDAC remains controversial; therefore, it is indispensable to identify powerful surrogate markers for predicting long survival after conversion surgery.

This research has some limitations. First, this was a single-institute, retrospective analysis with a limited number of cases. Second, the necessity of conversion surgery is debatable, even though CRT is successful. Patients receiving chemotherapy generally develop a tolerance to it, and surgical resection may be the most useful means of local control; however, there is no evidence to support this. In Japan, PREP-04 (UMIN000017793), which is a multi-institutional prospective observational study to investigate the effects of conversion surgery on patients with initial UR-PDAC, is ongoing. Given that only selected patients who responded favorably to non-surgical treatment were targeted among all patients with UR-PDAC, a selection bias existed. Conversion rates vary among reports on conversion surgery for patients with UR-PDAC. Nitshe et al. reported a conversion rate of 28.6% for FOLFIRINOX [29], while Hackert et al. reported that it was 50.8% [7]. Currently, FOLFIRINOX or gemcitabine plus nab-PTX is recommended for patients with UR-PDAC. It is still unclear which anticancer drug is optimal for conversion surgery. Moreover, the usefulness of adjuvant chemotherapy for resectable PDAC has been demonstrated in the JASPAC01 study, but the usefulness of adjuvant chemotherapy in conversion surgery has not yet been proven [10]. As for radiotherapy, it is controversial whether radiotherapy should be administered. Compared to resectable PDAC, the extent of surgical invasion for UR-PDAC is larger, and it is necessary to consider patients’ physical status, the drug used as adjuvant chemotherapy, and the administration period. Furthermore, the most relevant limitation of this study is represented by the lack of biological predictive markers that could support the selection of PDAC patients suitable for conversion surgery.

## 5. Conclusions

In conclusion, it is important to perform multidisciplinary treatment, including CRT with conversion surgery in patients with UR-PADC. However, many questions remain unsolved regarding the necessity of the conversion surgery including CRT, the optimal regimen, the duration of preoperative therapy, and criteria for surgical therapy. It is essential to perform prospective studies to resolve the various problems.

## Figures and Tables

**Table 1:** A comparison of the clinical characteristics between CS and non-CS patients.

Variables	Conversion Surgery		*p*-Value
	(+) (*n* = 20)	(−) (*n* = 378)	
age (years), median (range)	65 (44–83)	69 (33–87)	0.25
gender (M/F), *n*	(10/10)	(207/171)	0.656
tumor location (Ph/Pb,Pt), *n*	(14/6)	(218/160)	0.282
unresectability status (UR-LA vs UR-M), *n*	(9/11)	(243/135)	0.622
CEA, median (range) (U/mL)	3.4 (1.1–9.4)	4.0(0.3–845)	0.51
CA19-9, median (range) (U/mL)	2577 (0.6–1985)	211(0.6–50,000)	0.17
tumor size, median (range) (mm)	30 (18–50)	35(8–116)	0.323
T(4/3), *n*	(16/4)	(332/46)	0.385
N(1/0), *n*	(7/13)	(136/252)	0.917
M(1/0), *n*	(11/9)	(197/181)	0.829

M, male; F, female; CS, conversion surgery; Ph, pancreatic head; Pb, pancreatic body; Pt, pancreatic tail; UR-LA, unresectable locally advanced cancer; UR-M, unresectable cancer with metastasis. T, N, and M were classified according to the Tumor-Node-Metastasis (TNM) classification.

**Table 2:** Clinical characteristics of 20 patients who underwent conversion surgery.

Case	CEA (U/mL, Before)	CEA (U/mL, After)	CA19-9 (U/mL, Before)	CA19-9 (U/mL, After)	Tumor Size (mm, Before)	Tumor Size (mm, After)	Treatment Effect (RECIST)	Operative Method	Operative Time (min)	Bleeding Volume (mL)	Hospital Stay (Day)
1	2.4	2	25.9	9.9	27	13	PR	SSPPD	678	295	32
2	2.6	3.3	55.9	39	33	13	PR	DP	403	160	22
3	8	10.3	2789	24.9	32	23	PR	DP	373	260	50
4	1.1	5.1	81.4	34	50	50	SD	DP	438	1150	9
5	2.9	3.1	5103	19.9	23	12	PR	SSPPD + PVR	509	1250	11
6	9.4	4.4	92	10.9	29	13	PR	SSPPD	575	1220	23
7	6.4	2.4	253.5	49.6	24	9	PR	SSPPD + PVR	738	3010	16
8	2	2.7	9.2	11.4	42	10	PR	SSPPD	464	1030	10
9	1.5	1.6	1559	11.2	41	9	PR	SSPPD + PVR	574	1665	13
10	2.3	2.2	67.4	9	21	10	PR	DP	408	1075	11
11	3.4	3	20.6	7.4	30	16	PR	SSPPD + PVR	643	1390	9
12	2.5	3.2	1985	48.2	24	12	SD	SSPPD + HPR	841	2340	11
13	3.8	4.2	270	38.1	29	8	PR	SSPPD	729	2470	16
14	4.6	8	1515	16.4	41	34	PR	SSPPD + PVR	919	3600	47
15	5.1	3	1846	13.3	35	0	CR	DP	391	240	23
16	3.4	3.4	5.2	10.4	21	19	SD	DP	352	90	8
17	8	8.4	0.6	0.6	18	13	SD	SSPPD	623	1040	13
18	3	4.6	1047	10.7	32	11	PR	SSPPD	550	1020	8
19	1.8	2.1	516	17.8	30	9	PD	TP + PVR + HPR	613	460	14
20	2.9	9.5	330	99	24	25	SD	SSPPD + PVR	641	1695	13

UR-LA, unresectable locally advanced cancer; UR-M, unresectable cancer with metastasis; before, before initial therapy; after, after various therapy; PR, partial response; SD, stable disease; PR, partial response; SSPPD, substomach preserving pancretoduodenectomy; DP, distal pancreatectomy; PVR, portal vein resection; HPR, hepatic partial resection; TP, total pancreatectomy.

**Table 3:** Clinical characteristics of 20 patients who underwent conversion surgery. Each stage was classified according to the TNM classification.

Case	Stage (Before)	Stage (After)	p-Stage	Pathological	Evans
1	3	3	2a	tub2	IIb
2	3	2a	2a	tub1	I
3	3	3	2b	tub2	III
4	3	2b	2a	muc	IIb
5	3	2a	2a	tub1	IIb
6	3	1a	1	por	III
7	3	3	2b	tub1	III
8	3	3	2a	similar to NEN	IIb
9	3	3	1	tub2	IIb
10	4	3	0	no neoplasm	IV
11	4	2a	2a	tub1	III
12	4	2a	2a	tub2	I
13	4	2b	1	a few	III
14	4	4	2a	tub2	IIb
15	4	0	0	no neoplasm	IV
16	4	2a	2b	tub1	I
17	4	2a	1	tub1	III
18	4	3	2b	tub2	III
19	4	4	4	tub2	IIb
20	4	3	2a	tub2	I

UR-LA, unresectable locally advanced cancer; UR-M, unresectable cancer with metastasis; before, before initial therapy; after, after various therapy; *p*-stage, pathological stage; tub1, tubular adenocarcinoma with high differentiation; tub2, tubular adenocarcinoma with moderate differentiation; por, poorly differentiated adenocarcinoma; muc, mucinous adenocarcinoma; NEN, neuroendocrine neoplasm; a few, a few neoplasms; Evans, Evans classification.

**Table 4:** Predictive factors for the overall survival of patients with unresectable pancreatic cancer (univariate and multivariate logistic regression analyses).

Variables	Univariate Analysis			Multivariate Analysis		
	HR	95%CI	*p*-Value	HR	95%CI	*p*-Value
Age (per year)	0.67	-	0.54	-	-	-
Sex (male vs. female)	0.75	0.19–2.39	0.65	-	-	-
Location (Ph vs. Pb,Pt)	1.47	0.22–2.61	0.59	-	-	-
Tumor size (> 30 mm vs. <30 mm)	0.64	0.37–5.80	0.49	-	-	-
CEA (> 3 U/mL vs. <3 U/mL)	0.32	0.18–2.27	0.32	-	-	-
CA19-9 (> 100 U/mL vs. <100 U/mL)	0.62	0.15–1.88	0.45	-	-	-
UR-M vs. UR-LA	2.38	0.18–2.16	0.21	-	-	-
CRT ((+) vs.(−))	8.06	0.61–9.24	0.002	8.54	2.03–35.97	0.004
Tumor size (> 30 mm vs. <30 mm)	0.77	2.15–30.18	0.68	-	-	-
CEA > 3 U/mL vs. <3 U/mL)	1.23	0.22–2.69	0.76	-	-	-
CA19-9 (> 100 U/mL vs. <100 U/mL)	0.35	0.32–4.80	0.32	-	-	-
Change of tumor size (> 0.5 v.s. <0.5)	0.72	0.04–2.74	0.61	-	-	-
Change of CEA	1.5	0.20–2.55	0.56	-	-	--
Change of CA19-9	1	0.39–5.83	1	-	-	-
RECIST (PD,SD vs. PR,CR)	4.93	0.12–7.91	0.015	5.05	1.20-21.25	0.03
Period until operation (> 12 m vs. <12 m)	0.89	1.37–17.72	0.86	-	-	
Operation time (> 600 min vs. <600 min)	1.36	0.26–3.09 0.39–4.70	0.63	-	-	-
Bleeding volume (> 1000 mL vs. <1000 mL)	0.89	0.23–3.52	0.87	-	-	-
Evans (I-IIa vs. IIb-V)	0.57	0.12–2.76	0.49	-	-	-
pT (1,2 vs.3,4)	2.14	0.55–8.31	0.27	-	-	-
LN mets ((+) vs.(−))	0.77	0.16–3.67	0.75	-	-	-
Adjuvant chemo. ((−) vs(+))	2.07	0.53–8.10	0.29	-	-	-

HR, hazard ratio; CI, confidential interval; Ph, pancreatic head; Pb, pancreatic body; Pt, pancreatic tail; UR-LA, unresectable locally advanced cancer; UR-M, unresectable cancer with metastasis; CRT, chemoradiotherapy; PD, progressive disease; SD, stable disease; PR, partial response; CR, complete response; LN mets, lymph node metastasis.

## Data Availability

Not applicable
